# Association of Rotavirus Vaccination With Inpatient and Emergency Department Visits Among Children Seeking Care for Acute Gastroenteritis, 2010-2016

**DOI:** 10.1001/jamanetworkopen.2019.12242

**Published:** 2019-09-27

**Authors:** Daniel C. Payne, Janet A. Englund, Geoffrey A. Weinberg, Natasha B. Halasa, Julie A. Boom, Mary Allen Staat, Rangaraj Selvarangan, Parvin H. Azimi, Eileen J. Klein, Peter G. Szilagyi, James Chappell, Leila C. Sahni, Monica McNeal, Christopher J. Harrison, Mary E. Moffatt, Samantha H. Johnston, Slavica Mijatovic-Rustempasic, Mathew D. Esona, Jacqueline E. Tate, Aaron T. Curns, Mary E. Wikswo, Iddrisu Sulemana, Michael D. Bowen, Umesh D. Parashar

**Affiliations:** 1 Division of Viral Diseases, National Center for Immunization and Respiratory Diseases, Centers for Disease Control and Prevention, Atlanta, Georgia; 2 Department of Pediatrics, Seattle Children’s Hospital, Seattle, Washington; 3 Seattle Children’s Research Institute, Seattle Children’s Hospital, Seattle, Washington; 4 Department of Pediatrics, University of Rochester School of Medicine and Dentistry, Rochester, New York; 5 Department of Pediatrics, Vanderbilt University Medical Center, Nashville, Tennessee; 6 Immunization Project, Texas Children’s Hospital, Houston; 7 Department of Pediatrics, Baylor College of Medicine, Houston, Texas; 8 Division of Infectious Diseases, Cincinnati Children’s Hospital Medical Center, Cincinnati, Ohio; 9 Department of Pathology and Laboratory Medicine, Children’s Mercy Hospitals and Clinics, Children’s Mercy, Kansas City, Missouri; 10 Department of Infectious Disease, UCSF (University of California, San Francisco) Benioff Children’s Hospital Oakland, Oakland; 11 Department of Pediatrics, UCLA (University of California, Los Angeles); 12 Division of Infectious Diseases, Children’s Mercy, Kansas City, Missouri; 13 Division of Emergency Medicine, Children’s Mercy, Kansas City, Missouri

## Abstract

**Importance:**

Rotavirus vaccines have been recommended for universal US infant immunization for more than 10 years, and understanding their effectiveness is key to the continued success of the US rotavirus vaccine immunization program.

**Objective:**

To assess the association of RotaTeq (RV5) and Rotarix (RV1) with inpatient and emergency department (ED) visits for rotavirus infection.

**Design, Setting, and Participants:**

This case-control vaccine effectiveness study was performed at inpatient and ED clinical settings in 7 US pediatric medical institutions from November 1, 2009, through June 30, 2016. Children younger than 5 years seeking medical care for acute gastroenteritis were enrolled. Clinical and epidemiologic data, vaccination verification, and results of stool sample tests for laboratory-confirmed rotavirus were collected. Data were analyzed from November 1, 2009, through June 30, 2016.

**Main Outcomes and Measures:**

Rotavirus vaccine effectiveness for preventing rotavirus-associated inpatient and ED visits over time for each licensed vaccine, stratified by clinical severity and age.

**Results:**

Among the 10 813 children included (5927 boys [54.8%] and 4886 girls [45.2%]; median [range] age, 21 [8-59] months), RV5 and RV1 analyses found that compared with controls, rotavirus-positive cases were more often white (RV5, 535 [62.2%] vs 3310 [57.7%]; RV1, 163 [43.1%] vs 864 [35.1%]), privately insured (RV5, 620 [72.1%] vs 4388 [76.5%]; RV1, 305 [80.7%] vs 2140 [87.0%]), and older (median [range] age for RV5, 26 [8-59] months vs 21 [8-59] months; median [range] age for RV1, 22 [8-59] months vs 19 [8-59] months) but did not differ by sex. Among 1193 rotavirus-positive cases and 9620 rotavirus-negative controls, at least 1 dose of any rotavirus vaccine was 82% (95% CI, 77%-86%) protective against rotavirus-associated inpatient visits and 75% (95% CI, 71%-79%) protective against rotavirus-associated ED visits. No statistically significant difference during this 7-year period was observed for either rotavirus vaccine. Vaccine effectiveness against inpatient and ED visits was 81% (95% CI, 78%-84%) for RV5 (3 doses) and 78% (95% CI, 72%-82%) for RV1 (2 doses) among the study population. A mixed course of both vaccines provided 86% (95% CI, 74%-93%) protection. Rotavirus patients who were not vaccinated had severe infections 4 times more often than those who were vaccinated (74 of 426 [17.4%] vs 28 of 605 [4.6%]; *P* < .001), and any dose of rotavirus vaccine was 65% (95% CI, 56%-73%) effective against mild infections, 81% (95% CI, 76%-84%) against moderate infections, and 91% (95% CI, 85%-95%) against severe infections.

**Conclusions and Relevance:**

Evidence from this large postlicensure study of rotavirus vaccine performance in the United States from 2010 to 2016 suggests that RV5 and RV1 rotavirus vaccines continue to perform well, particularly in preventing inpatient visits and severe infections and among younger children.

## Key Points

**Question:**

Is rotavirus vaccination in the United States associated with a decrease in hospitalization and emergency department visits for rotavirus in children?

**Findings:**

This case-control vaccine effectiveness study included 1193 children with rotavirus infection and 9620 controls younger than 5 years with acute gastroenteritis in 7 US inpatient and emergency departments from 2010 to 2016. Both vaccines studied were associated with decreased inpatient visits and severe infections and among younger children.

**Meaning:**

These findings suggest that rotavirus vaccines continue to perform well, particularly among important target populations, continuing the justification of rotavirus vaccination in US infants.

## Teaser

This case-control study assesses US vaccine effectiveness for 2 rotavirus vaccines in preventing rotavirus inpatient and emergency department visits among children younger than 5 years.

## Introduction

The New Vaccine Surveillance Network (NVSN) has continuously monitored the effects and effectiveness of licensed rotavirus vaccines since the recommendation for universal US infant rotavirus vaccination by the Advisory Committee on Immunization Practices (ACIP).^1^ These live, attenuated, oral vaccines (RotaTeq [RV5; Merck & Co] and Rotarix [RV1; GlaxoSmithKline Biologicals]) performed well in prelicensure clinical trials^2,3,4,5^ and were recommended by the ACIP in 2006 and 2008, respectively.

During the last decade, as the ‎US rotavirus vaccination program has matured, precipitous decreases have been sustained in US hospitalizations^6,7,8^ and emergency department (ED) visits^9,10^ attributable to rotavirus gastroenteritis. However, this evidence of a public health effect may sometimes seem incongruous in relation to patient-level detections of rotavirus in children, particularly with the recent advent of highly sensitive multipathogen laboratory tests that potentially detect rotavirus shed from recently resolved infections and mild illnesses and even live-attenuated rotavirus vaccine virus shed naturally after immunization. Unbiased assessments of rotavirus vaccine effectiveness are important to maintain the public confidence in the US rotavirus immunization policy. Furthermore, rigorous US vaccine performance assessments inform and influence policy makers throughout the world on the initiation and continuation of their own rotavirus vaccination programs.

We conducted active rotavirus surveillance at 7 geographically diverse US pediatric medical institutions participating in the NVSN using a common protocol for participant enrollment, data collection, and laboratory confirmation from November 1, 2009, through June 30, 2016. To discern trends in rotavirus vaccine effectiveness during an extended period, we standardized and aggregated published NVSN data from 4 years (2010-2013)^11,12^ with previously unpublished NVSN data from 3 years (2014-2016). This accrual of comparable methods and case ascertainment across time allows an evaluation of rotavirus vaccine performance in the United States with large sample sizes, precision in results, and the ability to measure changes in rotavirus vaccine effectiveness over time.

## Methods

### Definition and Enrollment of Children

Active, prospective surveillance through NVSN is funded by the US Centers for Disease Control and Prevention (CDC), and methods have been previously published.^13^ Seven surveillance sites participated in this surveillance network, including Seattle Children’s Hospital (Seattle, Washington), University of Rochester School of Medicine and Dentistry (Rochester, New York), Monroe Carrell Jr Children’s Hospital at Vanderbilt (Nashville, Tennessee), Texas Children’s Hospital (Houston), Cincinnati Children’s Hospital Medical Center, (Cincinnati, Ohio), Children’s Mercy (Kansas City, Missouri), and UCSF (University of California, San Francisco) Benioff Children’s Hospital Oakland (Oakland). Institutional review board approval was obtained from the CDC and each study site, and all parents or guardians of participants provided written informed consent. Analysis and reporting of results were conducted in accordance with the Strengthening the Reporting of Observational Studies in Epidemiology (STROBE) reporting guidelines for case-control studies.^14^

Enrollment occurred from November 1, 2009, through June 30, 2010 (study year 2010), November 1, 2010, through June 30, 2011 (study year 2011), and December 1 through November 30, 2012, 2013, 2014, and 2015 (study years 2012-2015). Study year 2016 ranged from December 1, 2015, through June 30, 2016. USCF Benioff Children’s Hospital Oakland was included in all years except 2010 and 2012. These differences in enrollment were due to modifications to surveillance grant support during the study period.

The NVSN used common surveillance and laboratory protocols across all sites during the 2010 to 2016 period covered by this analysis. Children younger than 5 years were enrolled with informed consent from a parent or guardian on hospitalization or while visiting the ED with acute gastroenteritis (AGE), defined as diarrhea (≥3 episodes within 24 hours) and/or vomiting (≥1 episode within 24 hours). Exclusionary criteria involved indications of a noninfectious etiology, a history of immune deficiency, previous enrollment for the same AGE episode, or transfer from another hospital. Children who were originally enrolled in the ED but were subsequently hospitalized for the same illness were categorized as inpatients.

### Specimen Collection and Case Determination

Whole stool specimens were obtained within 10 days after AGE symptom onset, with more than 95% of specimens obtained within 7 days after onset. Surveillance sites first tested specimens using an enzyme immunoassay (EIA) (Premier Rotaclone; Meridian Bioscience, Inc). Specimens were shipped to the CDC, where rotavirus strains were genotyped using reverse transcription–polymerase chain reaction (RT-PCR) assays and nucleotide sequencing.^15^ Specimens that could not be genotyped were retested by EIA and real-time RT-PCR assay at the CDC. Any confirmed positive result led to a test-positive case designation. Specimens failing all CDC confirmatory analyses (<1%) were deemed to have indeterminate test results and were removed from the analytical data set.

Cases were defined as children with AGE symptoms who were hospitalized or treated in the ED and had a confirmed positive test result for rotavirus in the stool specimen during that AGE episode. Data from cases were compared with those from children with AGE whose specimens had negative test results for rotavirus by EIA (controls).

### Vaccine Effectiveness Analyses

Rotavirus immunization status was verified, as in previous publications,^11,12,13^ by obtaining vaccine records from primary care physicians or health care professionals and/or regional immunization information systems. Vaccine doses were defined as valid if given at least 14 days before onset of symptoms for the cases and controls. Children included in this study were required to be born on or after April 1, 2006, for RV5 analyses and on or after August 1, 2008, for RV1 analyses to ensure vaccine age eligibility following published ACIP recommendations.^1^ We restricted analyses to cases and controls who had reached the maximum ACIP-recommended age for completion of the vaccine series within the recommended age window (maximum age for the last dose, 8 months 0 days) to control for residual confounding by age at the time of last dose for both vaccine types.

We limited this analysis to children younger than 5 years across the 7-year study period. A test-negative case-control design was used to estimate the vaccine effectiveness, with cases defined as children age eligible to receive rotavirus vaccine who had a stool specimen with confirmed results positive for rotavirus and controls defined as children whose stool specimens had confirmed results negative for rotavirus. Vaccine effectiveness was calculated using the formula (1 − odds ratio) × 100 to estimate the preventive effect of rotavirus vaccines on rotavirus-associated hospitalizations and ED visits. Stratified vaccine effectiveness estimates were calculated by enrollment year, severity classification, age, vaccine product type, and vaccine dose number. A separate model was fit for each stratified estimate. The variables used in our models included surveillance site, month and year of birth, and month and year of enrollment. Adjusted odds ratios and 95% CIs were calculated by logistic regression and were adjusted for month and year of birth, month and year of symptom onset, and surveillance site. We calculated rotavirus vaccine effectiveness for those receiving at least 1 dose of a rotavirus vaccine by clinical setting over time, by severity classification, and by age, as well as by complete courses of RV5 only and RV1 only and by a mixed series of 3 doses of either RV5 or RV1 considered by the ACIP to constitute a complete course.^1^ We presented an aggregate vaccine effectiveness for any dose of either rotavirus vaccine type.

### Statistical Analysis

Data were analyzed from November 1, 2009, through June 30, 2016. We compared demographic and socioeconomic data for cases and controls using Wilcoxon rank sum tests for continuous variables and χ^2^ tests for categorical variables. Tests of statistical significance were 2-sided, and *P* < .05 was considered statistically significant. We assessed the clinical severity of AGE illnesses by calculating a modified Vesikari Severity Score (VSS)^12^ with severity classifications of mild (score ≤10), moderate (score 11-15), and severe (score ≥16) by clinical setting.

## Results

### Characteristics of Cases and Controls

Our vaccine effectiveness analysis included a total of 1193 laboratory-confirmed cases and 9620 controls, for a total of 10 813 participants (5927 boys [54.8%] and 4886 girls [45.2%]; median [range] age, 21 [8-59] months). By clinical setting, 360 cases (30.2%) were inpatients and 833 cases (69.8%) visited the ED. The RV5-specific, full-course vaccine effectiveness analysis included 860 rotavirus cases and 5736 controls. The RV1-specific, full-course vaccine effectiveness analysis included 378 rotavirus cases and 2461 controls (Table).

**Table.** Description of NVSN Participants Enrolled in RV5 and RV1 Analytical Data Sets^a^

Variables	RV5 Full-Course Analysis		*P* Value^b^	RV1 Full-Course Analysis		*P* Value^b^
	Cases With Rotavirus (n = 860)	Controls With AGE (n = 5736)		Cases With Rotavirus (n = 378)	Controls With AGE (n = 2461)	
Age, median (range), mo	26 (8-59)	21 (8-59)	<.001	22 (8-59)	19 (8-59)	<.001
Sex, No. (%)						
Male	464 (54.0)	3136 (54.7)	.69	213 (56.3)	1343 (54.6)	.52
Female	396 (46.0)	2600 (45.3)		165 (43.7)	1118 (45.4)	
Race, No. (%)						
White	535 (62.2)	3310 (57.7)	.01	163 (43.1)	864 (35.1)	.01
Black	215 (25.0)	1450 (25.3)		155 (41.0)	1097 (44.6)	
Other	110 (12.8)	976 (17.0)		60 (15.9)	500 (20.3)	
Ethnicity, No. (%)						
Hispanic	340 (39.5)	2489 (43.4)	.05	96 (25.4)	656 (26.7)	.86
Non-Hispanic	518 (60.2)	3242 (56.5)		282 (74.6)	1802 (73.2)	
Other or unknown	2 (0.2)	5 (0.1)		0	3 (0.1)	
Insurance, No. (%)						
Private	240 (27.9)	1348 (23.5)	.01	73 (19.3)	321 (13.0)	<.001
Public or none	620 (72.1)	4388 (76.5)		305 (80.7)	2140 (87.0)	
Clinical setting, No. (%)						
Inpatient	286 (33.3)	1151 (20.1)	<.001	125 (33.1)	448 (18.2)	<.001
ED	574 (66.7)	4585 (79.9)		253 (66.9)	2013 (81.8)	
Year, No. (%)						
2010	91 (10.6)	660 (11.5)	<.001	5 (1.3)	48 (2.0)	<.001
2011	205 (23.8)	689 (12.0)		51 (13.5)	84 (3.4)	
2012	50 (5.8)	821 (14.3)		15 (4.0)	297 (12.1)	
2013	218 (25.3)	964 (16.8)		84 (22.2)	434 (17.6)	
2014	81 (9.4)	1054 (18.4)		50 (13.2)	613 (24.9)	
2015	189 (22.0)	1021 (17.8)		152 (40.2)	683 (27.8)	
2016	26 (3.0)	527 (9.2)		21 (5.6)	302 (12.3)	
Site location, No. (%)						
Oakland, California	87 (10.1)	700 (12.2)	<.001	46 (12.2)	284 (11.5)	<.001
Seattle, Washington	94 (10.9)	658 (11.5)		19 (5.0)	85 (3.5)	
Kansas City, Missouri	127 (14.8)	692 (12.1)		157 (41.5)	1032 (41.9)	
Houston, Texas	329 (38.3)	1792 (31.2)		42 (11.1)	93 (3.8)	
Nashville, Tennessee	118 (13.7)	900 (15.7)		10 (2.6)	118 (4.8)	
Cincinnati, Ohio	62 (7.2)	533 (9.3)		74 (19.6)	644 (26.2)	
Rochester, New York	43 (5.0)	461 (8.0)		30 (7.9)	205 (8.3)	

Abbreviations: AGE, acute gastroenteritis; ED, emergency department; RV1, Rotarix; RV5, RotaTeq.

^a^ Percentages have been rounded and may not total 100.

^b^ Calculated using univariate *t* tests.

In the RV5 and RV1 analyses, rotavirus cases were more often white (RV5, 535 [62.2%] vs 3310 [57.7%]; RV1, 163 [43.1%] vs 864 [35.1%]), privately insured (RV5, 620 [72.1%] vs 4388 [76.5%]; RV1, 305 [80.7%] vs 2140 [87.0%]), and older (median [range] age for RV5, 26 [8-59] months vs 21 [8-59] months; median [range] age for RV1, 22 [8-59] months vs 19 [8-59] months) compared with controls, but did not differ by sex (Table). Vaccination status did not significantly differ by health insurance status, a potential proxy of health care–seeking behavior.

### Vesikari Severity Scores

We assessed clinical severity for 4 groups over time, comparing rotavirus infection status (cases vs controls) and whether rotavirus vaccine was received (any dose) for 1031 rotavirus cases and 8496 controls with full clinical severity data. Cases who were not vaccinated had infections of significantly greater severity compared with cases receiving at least 1 dose of rotavirus vaccine (median [range] VSS, 13 [3-19] vs 11 [2-18]). A severe VSS classification was assigned nearly 4 times as often for cases without vaccination compared with cases who were vaccinated (74 of 426 [17.4%] vs 28 of 605 [4.6%]). In comparison, controls had a median (range) VSS of 9 (2-19), regardless of vaccination status.

### Distribution of Rotavirus Cases by Time, Clinical Setting, and Age

**Figure 1.**
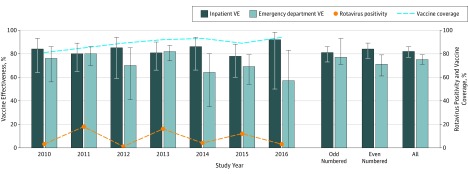
Rotavirus Vaccine Effectiveness (VE) Bar graph depicts effectiveness of at least 1 dose of vaccine in preventing inpatient or emergency department visits for acute gastroenteritis among children younger than 5 years, 2010 to 2016. Rotavirus positivity is depicted as percentage of inpatients admitted with acute gastroenteritis with positive results of testing for rotavirus. Rotavirus vaccine coverage was measured from the same population under active surveillance in Staat et al.^17^ Error bars indicate 95% CIs.

The introduction of rotavirus vaccines altered the historically annual peaks in US rotavirus incidence in favor of a new, biennial epidemiologic pattern of tempered incidence,^16^ a phenomenon also observed in a multicenter network of pediatric hospitals^17^ (Figure 1). Accordingly, the distribution of rotavirus cases in our vaccine effectiveness analysis also varied from year to year, with a higher incidence of cases occurring during odd-numbered years. During these odd-numbered years, rotavirus cases were older (median [range] age, 25 [8-59] months) compared with cases observed during even-numbered years (median [range] age, 23 [8-59] months among ED cases but similar [median {range} age, 26 {8-59} months] for both sets of years among the smaller sample of inpatients).

### Vaccine Effectiveness Results

Across the 7 years studied, a mean of 87% (median [range], 89% [79%-93%]) of all children enrolled in the NVSN had received any dose of rotavirus vaccine within the age window recommended by the ACIP (Figure 1). Similar to overall NVSN surveillance, 9195 (85.0%) of the subset of children included in our vaccine effectiveness analyses were vaccinated. Of the 1193 rotavirus cases in our analysis, 711 (60.0%) had received at least 1 dose of rotavirus vaccine. Of the 9620 controls, 8484 (88.2%) had been similarly vaccinated.

#### Vaccine Effectiveness for Those Receiving at Least 1 Dose of Rotavirus Vaccine

**Figure 2.**
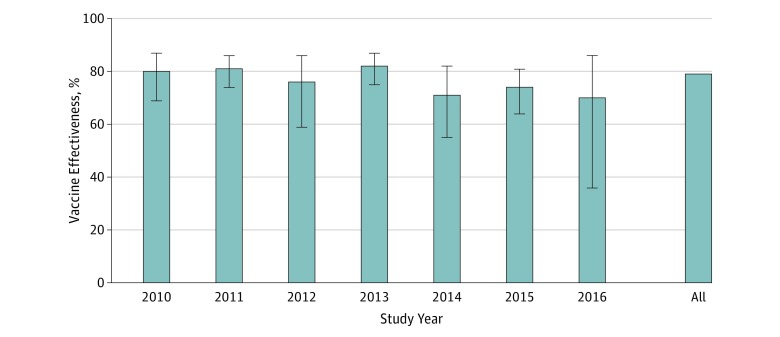
Aggregate Rotavirus Vaccine Effectiveness Graph depicts effectiveness of at least 1 vaccine dose in preventing inpatient or emergency department visits for acute gastroenteritis among children younger than 5 years, 2010 to 2016. Error bars indicate 95% CIs.

The 2010 to 2016 aggregate effectiveness of at least 1 dose of rotavirus vaccine to prevent a rotavirus-associated hospitalization or ED visit for AGE among US children younger than 5 years was 79% (95% CI, 76%-82%) (Figure 2). Stratified by clinical setting, any dose of rotavirus vaccine was 82% (95% CI, 77%-86%) protective against inpatient visits during these 7 years (Figure 1). Inpatient rotavirus vaccine effectiveness remained consistently high, ranging from 78% (in 2015) to 92% (in 2016), with no statistically significant downward trend detected over time for either rotavirus vaccine. Oscillations in inpatient vaccine effectiveness estimates were observed to occur biennially but were not statistically different, with 84% vaccine effectiveness in even-numbered years (95% CI, 76%-89%) compared with 81% vaccine effectiveness in odd-numbered years (95% CI, 73%-86%) (Figure 1).

Any dose of rotavirus vaccine was 75% (95% CI, 71%-79%) effective in preventing ED visits attributed to rotavirus gastroenteritis during these 7 years (Figure 1). Despite lower vaccine effectiveness point estimates for the ED clinical setting in the last 3 years of our study (including a nonsignificant ED vaccine effectiveness estimate for 2016), declines were not statistically significant for either rotavirus vaccine over time. Statistically nonsignificant oscillations in vaccine effectiveness estimates again occurred biennially in the ED clinical setting. However, in contrast to the inpatient trend, slightly higher vaccine effectiveness occurred in the ED during the odd-numbered years (77%; 95% CI, 71%-81%) compared with even-numbered years (71%; 95% CI, 61%-79%) (Figure 1).

**Figure 3.**
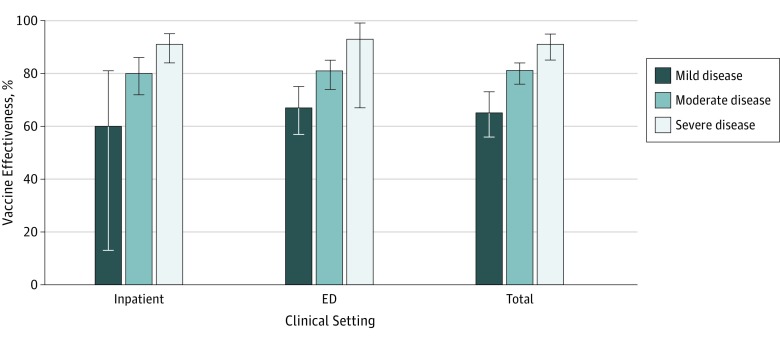
Rotavirus Vaccine Effectiveness by Disease Severity Classification and Clinical Setting Graph depicts effectiveness of at least 1 vaccine dose among children younger than 5 years, 2010 to 2016. Classification of disease severity is determined using the modified Vesikari Severity Score^12^ (mild indicates ≤10; moderate, 11-15; and severe, ≥16). Error bars indicate 95% CIs. ED indicates emergency department.

Because the VSS classification incorporates a severity value for hospitalization, we calculated vaccine effectiveness estimates separately for each clinical setting, as well as combined inpatient and ED estimates (Figure 3). A consistent gradation in vaccine effectiveness by severity existed, with vaccine protection greatest for more severe infections. In the combined clinical setting, any dose of rotavirus vaccine was 65% (95% CI, 56%-73%) effective against mild infections, 81% (95% CI, 76%-84%) against moderate infections, and 91% (95% CI, 85%-95%) against severe infections.

#### Vaccine Effectiveness for Full Courses of RV5 and RV1

**Figure 4.**
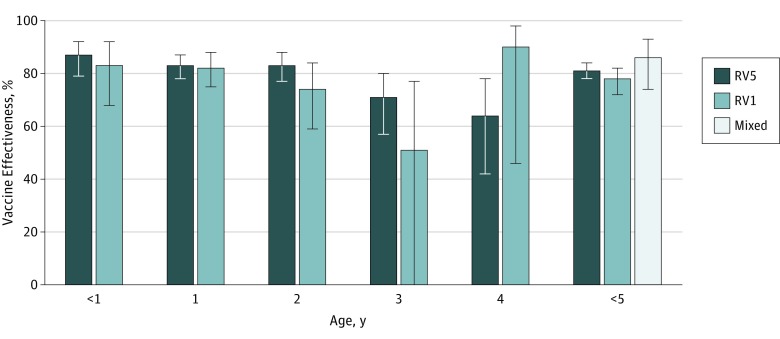
Rotavirus Vaccine Effectiveness for Complete Courses and Mixed Doses for Both Vaccines Graph depicts effectiveness in preventing inpatient and emergency department clinical visits for acute gastroenteritis by year of life, 2010 to 2016. RV1 indicates Rotarix; RV5, RotaTeq. Error bars indicate 95% CIs.

Among children younger than 5 years, the vaccine effectiveness against inpatient and ED visits was 81% (95% CI, 78%-84%) for RV5 and 78% (95% CI, 72%-82%) for RV1, and no statistical differences in vaccine performance were observed in this aggregate analysis (Figure 4). RV5 had its highest vaccine effectiveness in the first 3 years of life, ranging from 83% (95% CI, 77%-88%) to 87% (95% CI, 79%-92%). In the fourth and fifth years of life, RV5 protection was statistically lower than in the previous 3 years, at 71% (95% CI, 57%-80%) and then 64% (95% CI, 42%-78%), respectively (*P* = .008).

For RV1, vaccine effectiveness was similarly high in the first 3 years of life, ranging from 74% (95% CI, 59%-84%) to 83% (95% CI, 68%-92%). In the fourth year of life, protection was lower (51%; 95% CI, −6% to 77%) (*P* = .04), before rebounding to its highest level in the fifth year of life (90%; 95% CI, 46%-98%).

#### Vaccine Effectiveness for a Mixed Course of RV5 and RV1

We analyzed vaccine effectiveness for a mixed series of RV5 and RV1 vaccines pursuant to the ACIP recommendation that a child’s rotavirus vaccine series be completed with the same product whenever possible,^1^ but allowing interchanging vaccine types if the product used for a previous dose(s) is not available or is unknown. Among children younger than 5 years, the vaccine effectiveness of these mixed courses was 86% (95% CI, 74%-93%) against inpatient and ED visits (Figure 4).

## Discussion

Through 7 years of accumulated active surveillance results from geographically diverse pediatric medical institutions, our vaccine effectiveness assessment supports that RV5 and RV1 rotavirus vaccines continue to perform well in the United States and are associated with the prevention of inpatient visits, severe infections, and among younger children. Our findings suggest several important considerations regarding measurements of rotavirus vaccine effectiveness in the United States during the postlicensure era.

Rotavirus vaccine effectiveness was highest in preventing the most severe cases of rotavirus gastroenteritis, and even 1 dose of rotavirus vaccine appeared to be associated with less severe outcomes. Unvaccinated rotavirus cases were classified as having severe infections 4 times more often than vaccinated rotavirus cases. As with naturally acquired rotavirus exposures throughout childhood, which typically result in diarrheal episodes of decreasing severity, these live, attenuated rotavirus vaccines immunologically mimic an early exposure to rotavirus without causing symptomatic infection.^18^ Vaccination led to reduced clinical severity among participants enrolled in the early rotavirus vaccine efficacy trials^19,20^ and appears to remain true in the real-world setting of our phase 4 postlicensure assessment: RV1 and RV5 vaccines significantly reduce the clinical severity of rotavirus infections in childhood, and this finding alone is considered a successful outcome of vaccination.

Aggregate vaccine performance for RV5 and RV1 was statistically indistinguishable, and mixed courses of both vaccines appeared to be highly effective. Previously published RV5 (3-dose) vaccine effectiveness results from the NVSN for the first (range, 85%^11^ to 91%^12^), second (range, 82%^12^ to 90%^17^), third (range, 83%^11^ to 88%^12^), and fourth (range, 76%^12^ to 79%^11^) years of life were comparable to our findings. Our RV1 findings for these age groupings have greater study power and further clarify these previously published results. Earlier RV1 vaccine effectiveness estimates were previously derived from smaller samples, reflecting the delayed uptake of RV1 coverage across the United States.

We found that vaccine effectiveness for mixed-dose rotavirus vaccination course was 86% and was therefore potentially more protective, but this finding did not reach statistical significance. However, when added to previously published estimates (vaccine effectiveness, 80%),^21^ this further demonstrates that the estimated 100 000 US children who annually receive a mixed course of rotavirus vaccines are adequately protected. Although our data do not explain why a mixed course of rotavirus vaccines might be associated with higher overall protection than either vaccine alone, one hypothesis warranting further evaluation is whether receiving both vaccine types accrues specific immunologic benefits from each. Given the nonrandomized nature of our study, differences in estimated vaccine effectiveness might also be due to unmeasured, and thus unadjusted, confounding.

In longitudinal cohort studies conducted in Mexico City and Vellore, India,^22^ child age was associated with a decreased probability of further moderate to severe rotavirus infections. These cohort studies showed that rotavirus vaccine performance was associated with the age at which earliest infections occur. If applicable to US children, who are now born in an era of biennial oscillations in rotavirus incidence,^15^ the mean age of earliest rotavirus exposures may change depending on the annual birth cohort. Distinct annual birth cohorts of US children having differential probabilities in the age of first naturally acquired rotavirus exposures may potentially result in cohort-specific fluctuations in vaccine performance, a phenomenon that should not necessarily be equated with waning immunity.^23^ A further hypothesis involves the effect of season of birth on immunologic responses to vaccination,^24^ which may affect immunologic responses to vaccination. A longitudinal follow-up of specific birth cohorts in the postlicensure era may clarify these hypotheses. However, biennial oscillations in rotavirus incidence complicate our postlicensure-era understanding of long-term rotavirus vaccine performance in the United States.

Although rotavirus vaccine effectiveness was high among children during their earliest 3 years of life for both vaccines, fluctuations—but not always decreases—in vaccine effectiveness estimates were observed among older children. For instance, although we observed significantly lower RV1 protection in the fourth year of life, we provide the first US evidence that RV1 vaccine effectiveness in the fifth year of life (90%) was the highest of all studied age groups. Further mechanisms by which age influences protection against subsequent severe infections may include immune system maturity, greater likelihood of immunologic memory of prior rotavirus exposures, and lower likelihood of dehydration owing to a larger body mass and a greater ability to rehydrate independently.

Our study detected oscillations in inpatient and ED vaccine effectiveness estimates corresponding inversely with the biennial epidemiologic pattern of rotavirus incidence. In vaccine effectiveness analyses using pooled inpatient and ED data, these oscillations by clinical setting may be masked. Even-numbered years had the highest inpatient vaccine effectiveness (84% vs 81%), and odd-numbered years had the highest ED vaccine effectiveness (77% vs 71%) (Figure 1). We hypothesize that children who receive a vaccination and acquire a rotavirus infection of diminished severity or at an older age may be less likely to be hospitalized and may instead be rehydrated in the ED without escalating to inpatient care. Such a situation would have the epidemiologic effect that we observed in our calculations: A disproportionately higher concentration of patients with vaccine failures receiving care in the ED clinical setting would drive vaccine effectiveness down, and a lower concentration of cases who received vaccinations escalated to the inpatient setting would drive vaccine effectiveness up. This epidemiologic phenomenon could occur without any net increase in rotavirus cases overall.

These vaccine performance estimates have the highest statistical power yet published by the NVSN for RV5 and RV1. Furthermore, they include a geographically diverse sample that, when compared with the 2016 US bridged race postcensal estimates,^25^ adequately represent US children of black race and Hispanic ethnicity and reflects a conservative real-world estimate of rotavirus vaccine performance.

### Limitations

This study has limitations. Some test-negative case-control studies are affected by case misclassification and by differential health care–seeking behaviors. We have attempted to minimize the misclassification of cases with rotavirus through extensive additional laboratory confirmations. Our methods determined true rotavirus case status of children through EIA analyses, followed by confirmatory RT-PCR assays and nucleotide sequencing. With these laboratory confirmations, we estimate our laboratory results to have 100% sensitivity in detecting true rotavirus cases and 98.4% specificity, within the Rotaclone specificity estimate of 92% to 100%.^26^ Previous analyses on a subset of these data^11^ have indicated that the test-negative controls with AGE whom we used are the optimal control group for our design, despite the possibility of some residual differences in health care–seeking behavior.

## Conclusions

Our US rotavirus vaccine effectiveness estimates for 2010 through 2016 support the overall theme that RV5 and RV1 rotavirus vaccines perform consistently well, especially in the inpatient setting, with more severe infections, and among younger children. However, postlicensure-era dynamics in incidence, severity, and age pose challenges in interpreting vaccine effectiveness in a mature rotavirus vaccine program. Our analyses demonstrate that relying only on rotavirus test-positive surveillance data, without considering the underlying factors of age and clinical severity, could potentially misgauge the true measurements of rotavirus vaccine performance.

## References

[1] Cortese MM, Parashar UD; Centers for Disease Control and Prevention (CDC) Prevention of rotavirus gastroenteritis among infants and children: recommendations of the Advisory Committee on Immunization Practices (ACIP). MMWR Recomm Rep. 2009;58(RR-2):-. 19194371

[2] Ruiz-Palacios GM, Pérez-Schael I, Velázquez FR, ; Human Rotavirus Vaccine Study Group Safety and efficacy of an attenuated vaccine against severe rotavirus gastroenteritis. N Engl J Med. 2006;354(1):11-22. doi:10.1056/NEJMoa052434 16394298

[3] Linhares AC, Velázquez FR, Pérez-Schael I, ; Human Rotavirus Vaccine Study Group Efficacy and safety of an oral live attenuated human rotavirus vaccine against rotavirus gastroenteritis during the first 2 years of life in Latin American infants: a randomised, double-blind, placebo-controlled phase III study. Lancet. 2008;371(9619):1181-1189. doi:10.1016/S0140-6736(08)60524-3 18395579

[4] Vesikari T, Karvonen A, Puustinen L, Efficacy of RIX4414 live attenuated human rotavirus vaccine in Finnish infants. Pediatr Infect Dis J. 2004;23(10):937-943. doi:10.1097/01.inf.0000141722.10130.50 15602194

[5] Vesikari T, Karvonen A, Prymula R, Efficacy of human rotavirus vaccine against rotavirus gastroenteritis during the first 2 years of life in European infants: randomised, double-blind controlled study. Lancet. 2007;370(9601):1757-1763. doi:10.1016/S0140-6736(07)61744-9 18037080

[6] Shah MP, Dahl RM, Parashar UD, Lopman BA Annual changes in rotavirus hospitalization rates before and after rotavirus vaccine implementation in the United States. PLoS One. 2018;13(2):e0191429. doi:10.1371/journal.pone.0191429 29444124 PMC5812572

[7] Leshem E, Tate JE, Steiner CA, Curns AT, Lopman BA, Parashar UD National estimates of reductions in acute gastroenteritis-related hospitalizations and associated costs in US children after implementation of rotavirus vaccines. J Pediatric Infect Dis Soc. 2018;7(3):257-260. doi:10.1093/jpids/pix057 28992205

[8] Getachew HB, Dahl RM, Lopman BA, Parashar UD Rotavirus vaccines and health care utilization for diarrhea in US children, 2001-2015. Pediatr Infect Dis J. 2018;37(9):943-948. 29561514 10.1097/INF.0000000000001988 PMC7147954

[9] Rha B, Tate JE, Payne DC, Effectiveness and impact of rotavirus vaccines in the United States: 2006-2012. Expert Rev Vaccines. 2014;13(3):365-376. doi:10.1586/14760584.2014.877846 24392657 PMC12822838

[10] Shah MP, Tate JE, Steiner CA, Parashar UD Decline in emergency department visits for acute gastroenteritis among children in 10 US states after implementation of rotavirus vaccination, 2003 to 2013. Pediatr Infect Dis J. 2016;35(7):782-786. doi:10.1097/INF.0000000000001175 27088585 PMC5113824

[11] Payne DC, Boom JA, Staat MA, Effectiveness of pentavalent and monovalent rotavirus vaccines in concurrent use among US children <5 years of age, 2009-2011. Clin Infect Dis. 2013;57(1):13-20. doi:10.1093/cid/cit164 23487388 PMC4618548

[12] Payne DC, Selvarangan R, Azimi PH, Long-term consistency in US rotavirus vaccine protection: RV5 and RV1 vaccine effectiveness in US children, 2012-2013. Clin Infect Dis. 2015;61(12):1792-1799. doi:10.1093/cid/civ872 26449565 PMC7724934

[13] Payne DC, Staat MA, Edwards KM, Active, population-based surveillance for severe rotavirus gastroenteritis in children in the United States. Pediatrics. 2008;122(6):1235-1243. doi:10.1542/peds.2007-3378 19047240

[14] von Elm E, Altman DG, Egger M, Pocock SJ, Gøtzsche PC, Vandenbroucke JP; STROBE Initiative The Strengthening the Reporting of Observational Studies in Epidemiology (STROBE) statement: guidelines for reporting observational studies. J Clin Epidemiol. 2008;61(4):344-349. doi:10.1016/j.jclinepi.2007.11.008 18313558

[15] Bowen MD, Mijatovic-Rustempasic S, Esona MD, Rotavirus strain trends during the postlicensure vaccine era: United States, 2008-2013. J Infect Dis. 2016;214(5):732-738. doi:10.1093/infdis/jiw233 27302190 PMC5075963

[16] Aliabadi N, Tate JE, Haynes AK, Parashar UD; Centers for Disease Control and Prevention (CDC) Sustained decrease in laboratory detection of rotavirus after implementation of routine vaccination—United States, 2000-2014. MMWR Morb Mortal Wkly Rep. 2015;64(13):337-342. 25856253 PMC4584623

[17] Pindyck T, Tate JE, Parashar UD A decade of experience with rotavirus vaccination in the United States - vaccine uptake, effectiveness, and impact. Expert Rev Vaccines. 2018;17(7):593-606. doi:10.1080/14760584.2018.1489724 29909693 PMC9199965

[18] Velázquez FR, Matson DO, Calva JJ, Rotavirus infection in infants as protection against subsequent infections. N Engl J Med. 1996;335(14):1022-1028. doi:10.1056/NEJM199610033351404 8793926

[19] Vesikari T, Ruuska T, Delem A, André FE, Beards GM, Flewett TH Efficacy of two doses of RIT 4237 bovine rotavirus vaccine for prevention of rotavirus diarrhoea. Acta Paediatr Scand. 1991;80(2):173-180. doi:10.1111/j.1651-2227.1991.tb11830.x 1852084

[20] Clark HF, Borian FE, Bell LM, Modesto K, Gouvea V, Plotkin SA Protective effect of WC3 vaccine against rotavirus diarrhea in infants during a predominantly serotype 1 rotavirus season. J Infect Dis. 1988;158(3):570-587. doi:10.1093/infdis/158.3.570 2842405 PMC7110070

[21] Payne DC, Sulemana I, Parashar UD; New Vaccine Surveillance Network Evaluation of effectiveness of mixed rotavirus vaccine course for rotavirus gastroenteritis. JAMA Pediatr. 2016;170(7):708-710. doi:10.1001/jamapediatrics.2016.0014 27244539

[22] Lewnard JA, Lopman BA, Parashar UD, Naturally acquired immunity against rotavirus infection and gastroenteritis in children: paired reanalyses of birth cohort studies. J Infect Dis. 2017;216(3):317-326. doi:10.1093/infdis/jix310 28859432 PMC5853322

[23] Lewnard JA, Tedijanto C, Cowling BJ, Lipsitch M Measurement of vaccine direct effects under the test-negative design. Am J Epidemiol. 2018;187(12):2686-2697. doi:10.1093/aje/kwy163 30099505 PMC6269249

[24] Premkumar PS, Parashar UD, Gastanaduy PA, Reduced rotavirus vaccine effectiveness among children born during the rotavirus season: a pooled analysis of 5 case-control studies from the Americas. Clin Infect Dis. 2015;60(7):1075-1078. doi:10.1093/cid/ciu956 25452592 PMC7968047

[25] Centers for Disease Control and Prevention National Center for Health Statistics. Vintage 2016 Bridged-Race Postcensal Population Estimates. https://www.cdc.gov/nchs/nvss/bridged_race/data_documentation.htm#vintage2016. Published June 25, 2018. Accessed August 21, 2019.

[26] Premier Rotaclone [package insert]. Meridian Bioscience, Inc. http://www.meridianbioscience.com/uploads/696004_pi.pdf. Reviewed August 2018. Accessed August 21, 2019.

